# Hearing Loss in COVID-19 Patients: An Audiological Profile of Symptomatic and Asymptomatic COVID-19 Patients in Qatar

**DOI:** 10.7759/cureus.76326

**Published:** 2024-12-24

**Authors:** Reni K Chandran, Khalid Abdulhadi, Sarah Al-Shaikhly, Mohammed Ameen Arangodan, Nadeen Mousa Issa Ramadan, Shahed Jehad Ahmad Aldeeb, Brijesh Sathian

**Affiliations:** 1 Audiology and Balance Center, Hamad Medical Corporation, Doha, QAT; 2 Geriatrics and Long-Term Care, Rumailah Hospital - Hamad Medical Corporation, Doha, QAT

**Keywords:** asymptomatic covid, covid-19, distortion product otoacoustic emissions, hearing loss, symptomatic covid

## Abstract

Background and objective

Viral infections caused by cytomegalovirus, lymphocytic choriomeningitis virus, varicella-zoster virus, herpes simplex type 1 and type 2, rubella, measles, rubeola, HIV, West Nile virus, Lassa virus, and mumps are known to be associated with hearing loss. There have been reports of inner ear involvement in coronavirus disease 2019 (COVID-19) patients but the extent and variations in cochlear involvement of symptomatic and asymptomatic patients has not been adequately described. This study aimed to evaluate the hearing status among symptomatic and asymptomatic COVID-19 patients to address the prospects for routine screening for hearing loss in COVID-19 patients.

Methods

Patients testing positive for COVID-19 between March 2020 and May 2020 and August 2020 and October 2020 in Qatar were screened. A total of 110 patients aged 15-50 years were enrolled and grouped into symptomatic and asymptomatic COVID-19 after telephonic screening. Of them, seven were excluded for various reasons. A questionnaire was administered in person to all included participants. Audiological testing results of symptomatic and asymptomatic patients were analyzed.

Results

Of the 103 patients included in the study, 49 were symptomatic and 54 asymptomatic; 15 (14.6 %) had high-frequency sensorineural hearing loss (SNHL) in one or both ears. Mean thresholds in extended high frequencies 10K-20K were higher in symptomatic patients. Abnormal distortion product otoacoustic emissions (DPOAEs) were seen in 40 (38.8%) patients; 63.3% (31/49) were symptomatic and 16.7% (9/54) were asymptomatic in 3K-8K frequencies (p=0.0001).

Conclusions

Symptomatic COVID-19 patients had significant involvement of the inner ears with abnormal pure tone audiometry (PTA), extended high-frequency audiometry (EHFA), and DPOAEs compared to asymptomatic COVID-19 patients. The extent of inner-ear involvement suggests the severity of the infection. The lack of audiovestibular symptoms does not rule out normal hearing in such patients. Screening for hearing loss should be routinely considered in post-COVID-19 patients.

## Introduction

Viruses such as cytomegalovirus, lymphocytic choriomeningitis virus, varicella-zoster virus, herpes simplex type 1 and type 2, rubella, measles, rubeola, HIV, West Nile virus, Lassa virus, and mumps have been linked with hearing loss [1,2]. Severe acute respiratory syndrome coronavirus 2 (SARS-CoV-2), which causes coronavirus disease 2019 (COVID-19), has been isolated from the mastoid and middle ear, showing that it involves the respiratory epithelium of the middle ear and mastoid cells [3,4]. A study on the causal relationship between COVID-19 infection and audiovestibular dysfunction with human cellular models of infectious inner ear disease showed that human and mouse inner ear cells allow the entry of SARS-CoV-2; it can infect specific human inner ear cell types, suggesting that inner ear infection may underlie problems with hearing and balance in COVID-19 [5].

Case reports and systemic reviews have reported hearing loss associated with COVID-19 infection [6,7]. Acute as well as the long-term risks of coronavirus on audiovestibular systems warrant further analysis and description. The relationship between COVID-19 symptoms and sensorineural hearing loss (SNHL) appears to be complex, with cases reported in both symptomatic and asymptomatic individuals [6,7]. Further investigation, including large-scale prospective studies, is necessary to establish a definitive correlation between symptomatic COVID-19 infection and the probability of developing SNHL.

1.1. Hearing loss in viral infections and COVID-19

Hearing loss due to viral infections can be either unilateral, bilateral, sudden, or slowly progressive ranging from mild to profound. It is usually sensorineural, although it may be mixed or conductive [2]. A study on asymptomatic COVID-19-positive patients reported deleterious effects on the hair cells in the cochlea and demonstrated that the absence of the major symptoms does not guarantee a safe healthy cochlear function [8]. Another study involving 82 COVID-19 patients in Thailand reported one patient (1.22%) with neurosensory hearing loss [6]. A systematic review has suggested that high-quality studies are required in different age groups to investigate the acute effects of coronavirus, as well as understand long-term risks on the audiovestibular system [8].

The degree of involvement of the inner ear and middle ear dysfunction in COVID infection is yet to be described fully [5]. An individual infected with the SARS-CoV-2 virus can be symptomatic or asymptomatic (in terms of the known symptoms of COVID-19) and more than one-third of infections are known to be asymptomatic [9]. It is known that with cytomegalovirus infections, a leading non-genetic cause of childhood SNHL, permanent hearing loss occurs in both symptomatic and asymptomatic children [10,11]; however, the variations in the involvement of the inner ear between symptomatic and asymptomatic COVID-19 cases are not fully understood. Patients with and without any audiovestibular symptoms after COVID-19 were recruited for the study to assess their hearing and their subclinical cochlear function.

1.2. Symptomatic and asymptomatic COVID-19

By way of definition, a symptomatic COVID-19 case refers to a patient who has developed signs and symptoms such as sore throat, fever, loss of smell, cough, shortness of breath, and even gastrointestinal symptoms like diarrhea compatible with COVID-19 virus infection, which could be mild, moderate, or severe [12,13]. Patients with mild illness may exhibit a variety of signs and symptoms (e.g., fever, cough, sore throat, malaise, headache, muscle pain, nausea, vomiting, diarrhea, loss of taste and smell), with no shortness of breath, dyspnea on exertion, or abnormal imaging. In moderate illness, individuals show evidence of lower respiratory disease during clinical assessment or imaging and have an oxygen saturation (SpO_2_) ≥94% on room air at sea level. In severe illness, individuals have SpO_2_ <94% on room air at sea level, a respiratory rate >30 breaths/min, or lung infiltrates >50% [14]. The asymptomatic cases refer to true asymptomatic patients who never developed any COVID-19 symptoms. 

In this observational study, we explore the hearing status and audiological profile of symptomatic and asymptomatic COVID-19 PCR-positive patients treated at the Ambulatory Care Center, Audiology and Balance Unit, Hamad Medical Corporation, Qatar, by using tympanometry, acoustic reflexes, pure tone audiometry (PTA), high-frequency audiometry (HFA), and distortion product otoacoustic emission (DPOAE) tests.

## Materials and methods

A total of 265,978 patients tested positive for COVID-19 based on RT-PCR between March 2020 and May 2020 and August 2020 and October 2020 in Qatar. The initial treatment protocol involved hydroxychloroquine. A computerized random sample of 1,000 patients was generated from the 265,978 listed on the Communicable Disease Centre Infectious Disease Registry, and using documented medical history on Electronic Medical records (Cerner), and stratified as symptomatic and asymptomatic COVID-19 patients. In a telephonic screening, 421 were confirmed symptomatic and 579 were confirmed asymptomatic until the first 110 were reached. Asymptomatics consisted of only the true asymptomatic patients who had never developed COVID-19 symptoms (after excluding pre-symptomatic cases wrongly considered asymptomatics who became symptomatic later after the PCR test). Only those aged 15-50 years were included and patients with presbyacusis and congenital hearing loss, history of hearing loss, ototoxic medication, and noise exposure were excluded.

Telephone interviews started with a brief introduction explaining the purpose of this research study and our source of information about their COVID-19 infection. Following reassurance that the infection could affect some but not all of those infected, they were invited to visit the hospital for hearing tests, and appointments were scheduled. At the appointment, after signing informed consent, a questionnaire (with IRB approval/validity date) was administered; ears were examined by otoscopy followed by hearing tests (unless hard cerumen required removal; in such cases, a subsequent visit was scheduled). The 23 questions touched on demography, past noise exposure at the workplace, hearing loss or relatives with hearing loss, trauma to ears, history of meningitis, tinnitus, vertigo, the severity of COVID-19 illness, and comorbidities (see Appendices). No baseline audiograms, otoacoustic emissions (OAE), or other auditory assessments had been conducted before the patients' COVID-19 infection.

2.1. Sample size

Existing population-based studies of audiovestibular symptoms in confirmed COVID-19 cases constitute a few case reports characterized by a lack of precise primary outcomes [15], and hence could not be relied upon to compute a statistical sample size in this research study. Thus, 110 patients who met the inclusion criteria between 01/03/2020 and 31/05/2020, as well as 01/08/2020 and 31/10/2021, and were willing to participate were initially included.

2.2. Statistical analysis

The analysis was limited to rates, ratios, and proportions. Appropriate tests of significance were performed (test of proportions, chi-square, and Fisher's exact test). A two-sided p-value <0.05 was considered statistically significant. All statistical analyses were performed using SPSS Statistics 24.0 (IBM Corp., Armonk, NY) and Epi Info 2000 (Centers for Disease Control and Prevention, Atlanta, GA).

2.2. Test procedures

Hearing assessment, with immittance audiometry, PTA, HFA, and DPOAE tests, was done using calibrated GSI - Tympstar Pro, GSI- Audiostar Pro with TDH 50, GSI-Audiostar Pro DD450, and MADSEN Capella² with the OTO suite Otoacoustic Emissions module respectively. An ipsilateral stapedial at 0.5K, 1K, 2K, and 4K was elicited and considered normal if the elicited level was equal to or more than 0.03 mmho at an intensity between 80 and 100 dB. Hearing thresholds of 20 dBHL across the frequencies 250Hz-8K was considered normal. The HFA had no baseline thresholds but was compared between the asymptomatic and asymptomatic groups.

DPOAEs are responses generated by the cochlea to a two-tone stimulation presented to the ear to evaluate cochlear function. The preliminaries were fixed at L1=65dB SPL, and L2=55dB SPL with an f2/f1 ratio of 1.22. DPOAE testing was done at 500Hz, 1K, 2K, 3K, 4K, 5K, 6K, 7K, and 8K. The results were interpreted based on manufacturer settings [16] as follows: normal DPOAE: amplitude >5 dB and signal-to-noise ratio (SNR) >6; abnormal DPOAE: amplitude <5 dB and SNR >6; and absent DPOAE: amplitude <10 dB and SNR <6.

2.3. Ethical approval

This research study was approved on December 24, 2020, by the Institutional Review Board (IRB) of the Medical Research Center, Hamad Medical Corporation, Doha, Qatar (protocol number: MRC-01-20-903). It was funded and supported by the Medical Research Center (MRC), Hamad Medical Corporation.

## Results

From the 1000 random cases selected, 226 refused participation when called. Of the 110 enrolled, seven were excluded (for reasons such as taking hydroxychloroquine as part of treatment protocol or non-disclosure of ear trauma or meningitis). Hearing tests were done between 7-20 months post-infection (mean: 12.76 months). Among 103 COVID patients, 31 (30%) were Qataris, 72 (69.9%) were non-Qataris; 65 (63.1 %) were males 38 (36.9 %) were females; 49 (47. 6%) were symptomatic and 54 (52.4%) asymptomatic. The mean age of COVID patients was 34.23 ± 7.70 years. The mean age of males and females were 35.22 ± 6.5 and 32.55 ± 9.2 respectively; this difference was not statistically significant (t= -1.563, p=0.123). Forty-two (40.8%) and 36 (35%) cases were in the age groups of 25-34 and 35-44 years respectively; 14 (13.6%) patients were in the age group of 15-24 years while 11 (10.7%) were in the age group of 45-55 years.

The mean age of Qataris and non-Qataris was 30.68 ± 8.06 and 35.76 ± 7.07 years respectively; this difference was statistically significant (t= -3.208, p=0.002). The mean age of the symptomatic patients was 35 ± 7.8 years, while that of asymptomatic patients was 33.54 years ± 7.6, and the difference was not statistically significant (t=0.961, p=0.339). The self-reported audiovestibular symptoms among 103 patients (based on the questionnaire administered) showed that ear fullness, tinnitus, dizziness, and imbalance were seen in 28.6%, 24.5%, 22.4%, and 12.2% respectively among the symptomatic patients; the corresponding figures among asymptomatic patients were as follows: 1.9%, 5.6%, 5.6%, and 3.7% respectively. None of the study patients complained of hearing loss after COVID-19. Among the 49 symptomatic patients, 24 (49%) had one or more self-reported audiovestibular symptoms, while six (11%) of the 54 asymptomatic patients had one or more self-reported audiovestibular symptoms. This difference was significant (chi-sq.: 17.8, p<0.0001).

Among the 103 patients, 48 (46.6%) did not have self-reported audiovestibular symptoms or test abnormalities, while 24 (23.30%) with no self-reported audiovestibular symptoms had test abnormalities. Twelve (11.65%) of the 103 patients with self-reported audiovestibular symptoms had normal hearing tests and 19 (18.44%) with self-reported audiological symptoms had test abnormalities; this difference regarding the presence or absence of abnormal tests with or without self-reported symptoms was not significant (chi sq.: 1.22, p=0.26).

We observed that the frequency of SNHL in COVID patients increased with age (Table 1).

**Table 1:** Age group-wise comparison of SNHL in the cohort SNHL: sensorineural hearing loss

Age group, years	N (%)	SNHL, n (%)
15-24	14 (13.6)	1 (7.1)
25-34	42 (40.8)	3 (7.1)
35-44	36 (35)	6 (16.7)
>45	11 (10.7)	5 (45.5)
Total	103 (100)	15 (14.6)

3.1. Audiological assessment

3.1.1. Tympanometry

The results of tympanometry performed in 206 ears of COVID-19 patients are shown in Table 2.

**Table 2:** Tympanometry results between symptomatic and asymptomatic groups The chi-square test was the statistical test used and a p-value <0.05 is considered statistically significant

Tympanogram	Symptomatic, n (%)	Asymptomatic, n (%)	Chi-square	P-value
A type	89 (90.8%)	100 (95.2%)	1.54	0.214
Ad type	9 (9.1%)	5 (4.8%)		

One patient had type As and two patients had type C tympanogram recorded in the right ear and were not included in the comparison shown above which is based on the average values of left and right ears.

3.1.2. Pure Tone Audiometry (PTA)

Among 103 COVID-19 patients, 15 (14.6 %) had hearing loss on the PTA in one or both ears. Of these, 14 (93.3 %) were symptomatic and one (6.7 %) was asymptomatic. Among the 14 symptomatic patients, six (42.9%) had unilateral hearing loss and eight (57.1%) had bilateral hearing loss. Only one asymptomatic patient had bilateral hearing loss.

Hearing loss was classified as follows: mild high-frequency sensorineural loss with average thresholds of 4K, 6K, and 8K frequencies; mild sensorineural hearing loss with average thresholds of 500 Hz, 1K, 2K, and 4K; and mild mid and high-frequency sensorineural hearing loss of average thresholds of 2K, 4K, and 8K falling in the range of 21-40 dBHL.

Among the 49 symptomatic patients, mild high-frequency sensorineural hearing loss was seen in 10 (20.4%) patients in the right and left ears, one (2%) patient had mild sensorineural hearing loss, and one ( 2%) patient had mild mid and high-frequency sensorineural hearing loss in the right ear. Among the 54 asymptomatic patients, one patient (1.9%) had mild high-frequency sensorineural hearing while the rest (98.1%) had normal hearing.

3.1.3. Extended High-Frequency Audiometry (EHFA)

The mean thresholds in the high frequencies of 10 K-20 K were higher in the symptomatic group compared to the asymptomatic group, as shown in Table 3.

**Table 3:** Mean threshold trends in high-frequency audiometry (10-20 kHz) between the groups The t-test was the statistical test used and a p-value <0.05 is considered statistically significant COVID-19: coronavirus disease 2019; EHFA: extended high-frequency audiometry

	COVID-19 symptoms	N	Mean threshold	Standard deviation	t-value	P-value
Right EHFA 10K	Symptomatic	49	17.04	12.20	2.06	0.04
	Asymptomatic	54	12.31	11.06		
Right EHFA 12.5K	Symptomatic	49	24.69	21.07	1.62	0.10
	Asymptomatic	54	18.70	16.11		
Right EHFA 16K	Symptomatic	49	36.84	16.95	0.26	0.79
	Asymptomatic	54	35.93	18.30		
Right EHFA 20K	Symptomatic	49	21.84	4.02	5.01	< 0.001
	Asymptomatic	54	17.69	4.37		
Left EHFA 10K	Symptomatic	49	17.65	14.10	1.44	0.15
	Asymptomatic	54	13.89	12.07		
Left EHFA 12.5K	Symptomatic	49	24.18	21.12	1.71	0.09
	Asymptomatic	54	17.87	16.18		
Left EHFA 16K	Symptomatic	49	35.10	17.64	0.63	0.52
	Asymptomatic	54	32.78	19.16		
Left EHFA 20K	Symptomatic	49	21.53	5.45	2.99	0.003
	Asymptomatic	54	18.70	4.09		

3.1.4. Diagnostic distortion product Otoacoustic emission (DPOAE)

**Figure 1:**
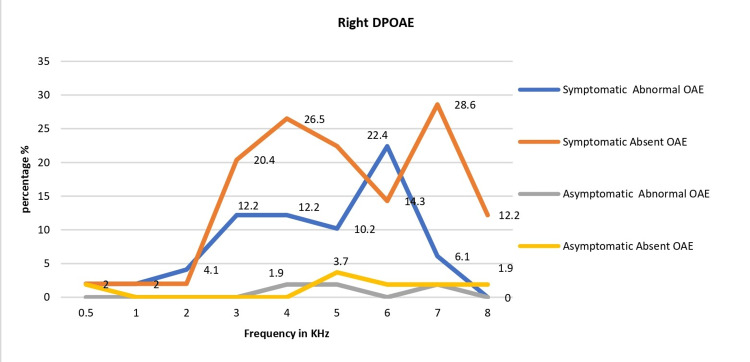
Right-ear DPOAE: frequency-specific response patterns DPOAE: distortion product otoacoustic emission

**Figure 2:**
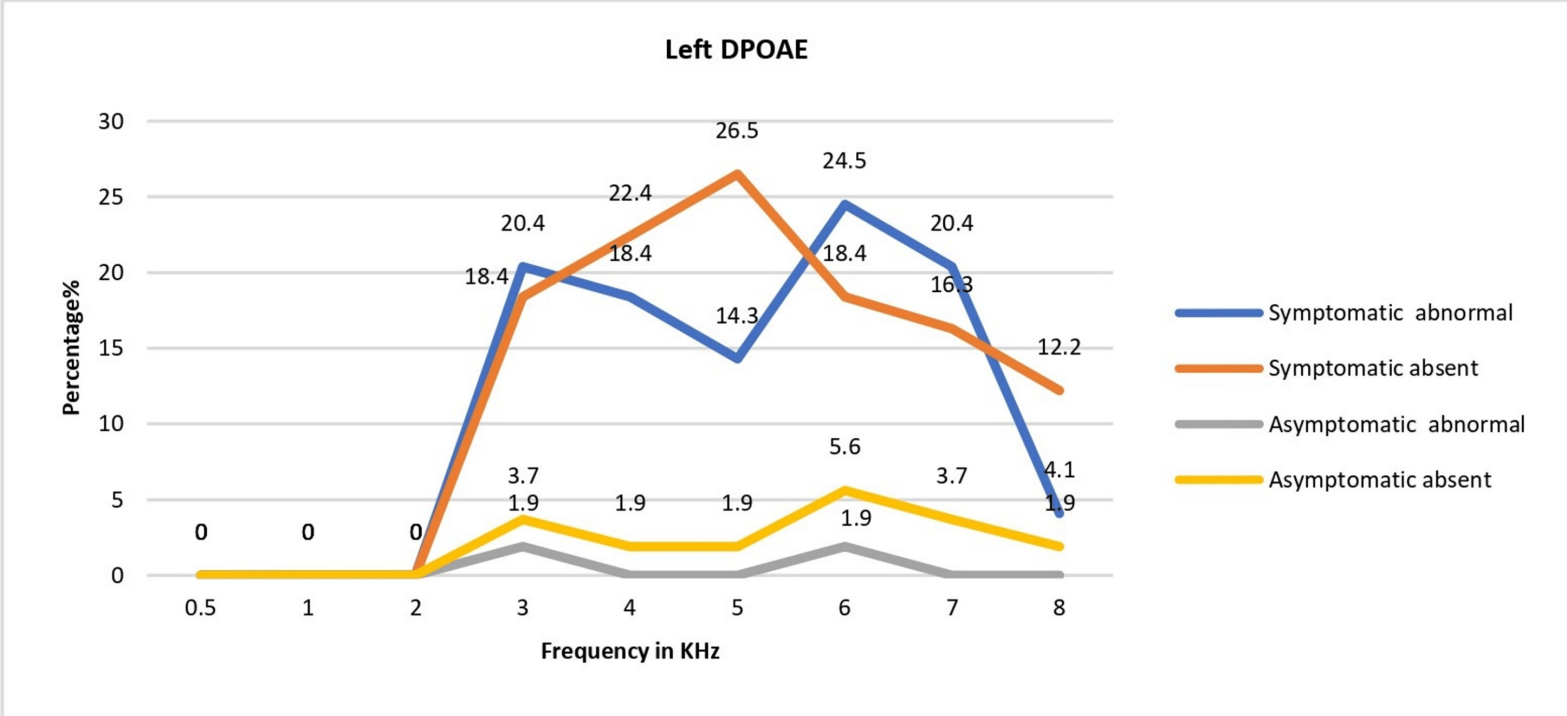
Left-ear DPOAE: frequency-specific response patterns DPOAE: distortion product otoacoustic emission

Based on the DPOAE test, of the 103 patients, 40 (38.83%) patients had abnormal DPOAE amplitude; 63.3% (31/49) were symptomatic and 16.7% (9/54) were asymptomatic. This difference was significant (chi-sq.: 23.58, p=0.0001). The absent and abnormal DPOAE responses were significantly higher in the symptomatic than in the asymptomatic group in both right and left ears (Figures 1-2).

The amplitudes of the DPOAEs were abnormal predominantly in 3K-8K frequencies, sparing the low frequencies, and the abnormal amplitudes were significantly higher in the symptomatic group than in the asymptomatic group (Table 4). 

**Table 4:** Comparison of frequency-based DPOAE amplitudes between symptomatic and asymptomatic COVID-19 patients The chi-square test was the statistical test used and a p-value <0.05 is considered statistically significant COVID-19: coronavirus disease 2019; DPOAE: distortion product otoacoustic emission

Frequencies	Group	Abnormal DPOAE amplitude, n (%)	Normal DPOAE amplitude, n (%)	Chi-square value	P-value
500Hz	Symptomatic	0	49 (100)	NA	NA
	Asymptomatic	0	54 (100)		
1K	Symptomatic	1 (2)	48 (98)	0.002	0.961
	Asymptomatic	0	54 (100)		
2K	Symptomatic	1 (2)	48 (98)	0.002	0.961
	Asymptomatic	0	54 (100)		
3K	Symptomatic	22 (44.9)	27 (55.1)	21.633	<0.0001
	Asymptomatic	3 (5.6)	51 (94.4)		
4K	Symptomatic	22 (44.9)	27 (55.1)	24.393	<0.0001
	Asymptomatic	2 (3.7)	52 (96.3)		
5K	Symptomatic	24 (49)	25 (51)	25.044	<0.0001
	Asymptomatic	3 (5.6)	51 (94.4)		
6K	Symptomatic	21 (42.9)	28 (57.1)	22.706	<0.0001
	Asymptomatic	2 (3.7)	52 (96.3)		
7K	Symptomatic	22 (44.9)	27 (55.1)	27.446	<0.0001
	Asymptomatic	1 (1.9)	53 (98.1)		
8K	Symptomatic	7 (14.3)	42 (85.7)	5.544	0.019
	Asymptomatic	1 (1.9)	53 (98.1)		

Among 15 COVID-19 patients with tinnitus, 20% (3/15) had a hearing loss on PTA testing; of these, two (67%) were symptomatic and one (33%) was asymptomatic. Whereas out of 15 patients with tinnitus, 11 (73.3%) had abnormal DPOAE responses; 10 (90.9%) were symptomatic and one (9.1%) was asymptomatic. No significant statistical difference was noted when comparing the abnormal amplitudes of DPOAE responses on the right and left ears between symptomatic and asymptomatic patients with tinnitus. Associated comorbidities like diabetes mellitus and hypertension were seen in nine out of the 103 COVID-19 PCR-positive patients. Of these, four were symptomatic and five were asymptomatic. Three of the four symptomatic patients had a hearing loss on PTA testing and all four symptomatic comorbid COVID-19 patients had abnormal DPOAE test results; however, none in the comorbid asymptomatic group had a hearing loss on PTA while one had abnormal DPOAE tests results.

## Discussion

This study sheds light on the involvement of the auditory system in COVID-19 infection and the variation in the audiological symptoms between symptomatic and asymptomatic patients. Among symptomatic patients, 24 (49%) had one or more self-reported audiovestibular symptoms, and aural fullness was the most common, as reported in several other studies [17, 18]. However, among asymptomatic patients, tinnitus and dizziness were more prevalent than aural fullness. Of the 103 patients, 24 (23.30%) with no self-reported audiovestibular symptoms had hearing test abnormalities, indicating that the absence of audiovestibular symptoms does not guarantee a normal inner ear function.

The middle ear status as measured by tympanometry showed normal middle ear status in 89% of the patients, and none had features of middle ear effusion. This could be attributed to the fact that testing was done around one year post-COVID-19 when the chances for acute or subacute middle ear effusion would have resolved. Among 103 COVID-19 patients, 14.56% had hearing loss on PTA in the high frequencies in one or both ears. In a study involving 82 COVID-19 patients from Thailand, one patient (1.22%) had neurosensory hearing loss [6]. A review of the literature showed a dearth of details regarding audiovestibular symptoms and a lack of data on the type or severity of hearing loss in many of the case reports and cross-sectional studies [7]. 

Another study [5] indicated that all 10 symptomatic COVID-19 patients included in their analysis had audiovestibular involvement, with the majority presenting with significant hearing loss. However, the study did not clarify whether these individuals' COVID-19 symptoms were mild, moderate, or severe. This lack of data makes it difficult to assess if the level of hearing loss is related to the severity of the illness. Furthermore, the study did not specify whether the hearing loss was temporary or permanent, nor did it take into account other relevant factors such as patient demographics, pre-existing hearing disorders, or comorbidities. This absence emphasizes the need for more studies to better understand the association between COVID-19 severity and audiovestibular impairment.

Our study mainly assessed COVID-19 patients with mild to moderate symptoms; those with severe COVID-19 were apprehensive about getting reinfected during hospital visits during the second wave of the pandemic in Qatar. Some others were recovering from other systemic illnesses associated with COVID-19. Our study had only one severe case of COVID-19, a 46-year-old female, admitted to the ICU and had noninvasive ventilation for six days with a saturation drop of 93% at admission. Even though she had no prior hearing loss complaints, her hearing test indicated a slight high-frequency SNHL, with bilateral DPOAEs affected. Comorbid diseases and treatment ototoxicity, such as antivirals, antibiotics, or corticosteroids, may all contribute to severe or abrupt SNHL in severe COVID-19 patients [19]. High-frequency hearing loss (4-8 KHz) was the most prevalent type in our study, similar to prior studies reporting higher thresholds at high frequencies among COVID-19 patients compared to controls [20]. Notably, DPOAE abnormalities were most common between 3 and 8 KHz, sparing lower frequencies, indicating that the mid and high frequencies are most sensitive.

This trend might be attributed to the anatomical and metabolic properties of the cochlear basal turn, which processes high frequencies and is more vulnerable to viral infections, hypoxia, and vascular compromise - all of which are frequent in severe COVID-19 [8]. Furthermore, ototoxic medicines, such as hydroxychloroquine, have a disproportionate influence on high-frequency hearing because of their unique effect on basal cochlear hair cells [17]. SARS-CoV-2's direct involvement in inner ear infections compounds this susceptibility since the virus has been demonstrated to infect and harm cochlear cells [5]. The causes for frequency-specific vulnerability remain unknown, emphasizing the need for more studies, especially longitudinal studies, to discover if these effects are temporary or persistent. Understanding these pathways is critical for improving post-COVID-19 audiological care and designing targeted therapies to prevent or minimize high-frequency cochlear damage.

A large proportion of those with no self-reported audiovestibular symptoms had test abnormalities; hence, post-COVID-19 screening for hearing loss should be routinely performed and those with tinnitus should have DPOAE in addition to PTA since it appears to be able to identify more abnormalities. Likely, we may not be able to conduct future studies among those with severe COVID-19 symptoms because of the disappearance of the earlier strains causing more severe illnesses.

Limitations 

This study has a few limitations. No baseline audiograms, otoacoustic emissions (OAE), or other auditory testing were conducted before COVID-19 infection. The absence of pre-exposure data impedes the determination of whether the reported hearing abnormalities were pre-existing conditions or directly induced by the illness. Moreover, the study's reliance on patient self-reports for prior auditory health introduces the potential for recall bias, thereby confounding the interpretation of findings. Carrying out this research during the lockdown was a challenge. While we started with a list of 1000 random cases, the first 110 who were willing to participate were selected for our study, and those with severe infection were excluded because of age and potential ototoxic medications. Hence, these results cannot be extrapolated to the general population but can only be compared to similar groups with mild and moderate COVID-19 symptoms.

Pre-covid baseline hearing test results were not available for comparison, and nor was it possible to design a study that included controls for a similar comparison. Although we assumed that symptomatic patients would have worse hearing outcomes than asymptomatic patients, it is possible that some of the participants had a hearing deficit before their COVID-19 infection and there was no way to adjust for this. The process of identifying truly asymptomatic COVID-19 patients was cumbersome, which led us to extend the duration of the study period. The vestibular involvement was not assessed although some patients had symptoms of dizziness.

## Conclusions

Symptomatic COVID-19 patients had significant involvement of the inner ears compared to asymptomatic patients. The absence of audiovestibular symptoms cannot ensure normal hearing in COVID-19 patients, especially in the symptomatic group. Hence, screening for hearing loss and cochlear involvement in COVID-19 patients should be routinely conducted. COVID-19 tends to involve mid and high-frequency cochlear hair cells. Significantly abnormal DPOAE test results and PTA and EHFA test results were seen in symptomatic patients compared to asymptomatic ones, suggesting that the extent of the involvement of the inner ear is proportionate to the severity of infection. The pathogenicity of symptomatic and asymptomatic COVID-19 is not yet fully understood to form further inferences. DPOAE test would be a better tool to assess the cochlear status than PTA in COVID-19 patients complaining of tinnitus. Coexisting comorbidities like diabetes and hypertension had a higher association with high-frequency hearing loss.

## Appendices

**Table 5:** Study questionnaire

Questionnaire
Gender
Male
Female
Your age, years
15-25
26-35
36-45
46-50
What is your job?
Are you exposed to loud sounds at your workplace?
Yes
No
Have you been exposed to loud sounds for a prolonged period of time?
Yes
No
Have you been diagnosed with hearing loss since birth?
Yes
No
Do you have any relatives diagnosed with hearing loss?
Yes
No
If yes, who and how many?
Do you have any history of head trauma or trauma to your ears?
Yes
No
Have you been diagnosed with meningitis?
Yes
No
Do you have any history of hyperbilirubinemia?
Yes
No
Do you have hearing loss?
Yes
No
If yes, then
Right ear
Left ear
Both?
Do you have an ear block sensation?
Yes
No
If yes, then
Right ear
Left ear
Both
Do you have ringing sounds in your ear?
Yes
No
If yes, then
Right ear
Left ear
Both
Do you have dizziness after the COVID-19 infection?
Yes
No
If yes, then
Rotatory
Nonrotatory
Light-headedness
Do you have an imbalance?
Yes
No
Do you have an intolerance to loud sounds after the COVID-19 infection?
Yes
No
Did you have a fever with COVID-19 infection?
Yes
No
Did you have a sore throat with the COVID-19 infection?
Yes
No
Did you have breathing difficulty with the COVID-19 infection?
Yes
No
Were you admitted to the hospital for COVID-19?
Yes
No
Were you admitted to the ICU for COVID-19?
Yes
No
If yes, then mechanical ventilation
Yes
No
Do you have any comorbidities?
Yes
No
If yes,
Diabetes
Hypertension
Dyslipidemia
Coronary artery disease
